# Core competencies for a biomedical laboratory scientist – a Delphi study

**DOI:** 10.1186/s12909-022-03509-1

**Published:** 2022-06-20

**Authors:** Maria M. Stollenwerk, Anna Gustafsson, Gudrun Edgren, Petri Gudmundsson, Magnus Lindqvist, Tommy Eriksson

**Affiliations:** 1 Department of Biomedical Science, Faculty of Health and Society, Malmö University, Malmö, Sweden; 2 Biofilms Research Center for Biointerfaces, Malmö University, Malmö, Sweden; 3 Center for Teaching and Learning, Faculty of Medicine, Lund University, Lund, Sweden; 4 University Executive Office, Malmö University, Malmö, Sweden

**Keywords:** Core curriculum, Core competencies, Delphi, Biomedical laboratory scientists, Student, Expert group, Biomedical laboratory scientist degree

## Abstract

**Background:**

After completing university education, biomedical laboratory scientists work in clinical laboratories, in biomedical research laboratories, in biotech, and in pharmaceutical companies. Laboratory diagnostics have undergone rapid development over the recent years, with the pace showing no signs of abatement. This rapid development challenges the competence of the staff and will most certainly influence the education of future staff. This study aimed to examine what was considered the necessary competencies needed to pursue a career as a biomedical laboratory scientist.

**Methods:**

A modified Delphi technique was used, with the panel of experts expressing their views in a series of three questionnaire. Consensus was defined as the point which 75 % or more of the panel participants agreed that a particular competency was necessary.

**Results:**

The study highlights the perceived importance of mostly generic competencies that relate to quality, quality assurance, and accuracy, as well as different aspects of safety, respect, trustworthiness (towards patients/clients and colleagues), and communication skills. The results also stress the significance of self-awareness and professionality.

**Conclusions:**

We identified important competencies for biomedical laboratory scientists. Together with complementary information from other sources, i.e., guidelines, laws, and scientific publications, the competencies identified can be used as learning outcomes in a competency-based education to provide students with all the competencies needed to work as professional biomedical laboratory scientists.

**Supplementary Information:**

The online version contains supplementary material available at 10.1186/s12909-022-03509-1.

## Introduction

In modern health care, laboratory analyses are essential tools in securing diagnoses and treatment follow-up. Yet, such laboratory activities are by large hidden from the public, decision makers, and politicians. However, the need for testing and sample analyses during the Covid-19 pandemic has led to increased attention to laboratory diagnostics as a fundamental element of the health system. Organization of laboratories and required qualifications of laboratory staff vary internationally. In some countries, the staff are typically licensed with university degrees in (bio) medical laboratory science, while in other countries the requirements are less rigorous. In some countries, laboratory medicine is (also) a medical specialty, with an individual with a medical degree usually overseeing the laboratories. Elsewhere, heads of laboratories are more likely to be biochemists, usually with a Ph.D. degree.

Biomedical laboratory scientists work in clinical laboratories (e.g., immunology, pathology, microbiology, transfusion medicine, or physiology), biomedical research laboratories, and biotechnology and pharmaceutical companies. They perform a range of laboratory assays on tissue samples, blood, and body fluids, which are crucial to the clinician’s work in forming diagnoses and treatment protocols. In clinical physiology, the staff perform a range of diagnostic examinations in close contact with patients. The continuing expansion in biomedical knowledge, the rapid development of methodology and technology, and the increased involvement of automation are likely to significantly impact the demands placed on the education of future biomedical laboratory scientists.

Outcome-based and competency-based education (CBE) has attracted increased attention over recent decades, particularly in health sciences education [1–3]. The focus of education has changed from passing time-based courses with varying results between individuals to a focus on learning outcomes to be achieved by all students, thus potentially addressing accountability issues [2]. Health care systems are changing rapidly due to increasing demands from the public regarding availability and equity. Consequently, education of healthcare professionals needs to respond to these changes. Outcome-based education, where outcomes are defined as competencies, holds promises as a viable alternative to a more conventional approach [4]. The competencies defined for the education of a specific health profession need to be determined to meet the requirements of both the public and the health care system. Furthermore, the education needs to be in alignment with the mission and vision of the educational institution. Assessment is an essential part of CBE employed to ascertain that all graduates meet the defined outcomes. It should include not only traditional methods for evaluation of knowledge and skills, but also competencies in practice. Feedback during the learning process is important for the professional development of individual learners [2]. Focusing on the attainment of learning outcomes means that different learners may not need the same time for learning; thus, the training may vary [4]. However, since this is often not in agreement with national regulations, time variability can instead be used for elective courses or research experience. In particular, frameworks for CBE for health professions have been developed for medicine for both undergraduate and postgraduate education. Er et al. [5] refer to the implementation of CBE at a number of health education programs at a university in Malaysia. A well-known example is the Canadian CanMEDs framework [2, 6]. Other health professions have shown less interest in CBE, judging by the number of research publications, with the exception of nursing and pharmacy [7, 8].

Except for Edgren, 2006 [9], we have not found any publications on CBE in biomedical laboratory scientist educational programmes, but a couple on competencies and curricula for the similar clinical laboratory science [10, 11]. The International Federation of Biomedical Laboratory Science (IFBLS) has issued guidelines on core competencies and core curricula for biomedical laboratory scientist/biomedical scientists [12]. However, examples contained in the guidelines are both detailed and subject-based, and thus not in agreement with the CBE approach. The EPBS (European Association for Professions in Biomedical Science) [13] has only the following recommendation on education outcomes: minimum standard for entry into the biomedical science profession in Europe is EQF Level 6 (European Qualification Framework) [14], i.e. bachelor level or 1st cycle (180 - 240 ECTS) under the Bologna Process. In contrast to the IFBLS core competencies, this competency level includes knowledge and skills, but the proposed qualification is comprehensive and generic; thus it is not sufficient to define a CBE curriculum. We have found numerous studies on curriculum planning and core competencies for other health professions, mainly nursing, pharmacy, dentistry and medicine [15–36].

Education planning thus involves identifying what is relevant for good professional practice (learning outcomes), forming the basis for course outcomes, for teaching and learning, and for examination [2, 37]. Developing a new core curriculum to match the competencies needed for the professional activities of biomedical laboratory scientists is important to meet the challenges of the medical/health care sector.

The Delphi technique, developed by the Rand Corporation in the USA in the 1950s as a technique to foresee future events, was chosen for this study. The Delphi technique is based on statements from a panel of experts in the field to be developed. The panel expresses its views in a series of questionnaires until consensus is achieved [38–41]. In particular, the technique has been used extensively for the development of education, but there only are a handful of reviews on the approach [38, 40, 42, 43]. Although several different methods can be used (see, e.g., Dunn et al. [44]), we consider the Delphi technique the most suitable based on a literature review and on local experiences in the training of biomedical scientists, pharmacists, physicians, and ambulance nurses [8, 9, 45, 46].

This study aimed to identify competencies deemed necessary for employment of a newly qualified biomedical laboratory scientist. Specifically, looking at a five-year perspective, we wanted to study the competencies a future colleague or an employer might be expecting from a recently graduated biomedical laboratory scientist.

## Methods

**Fig. 1:**
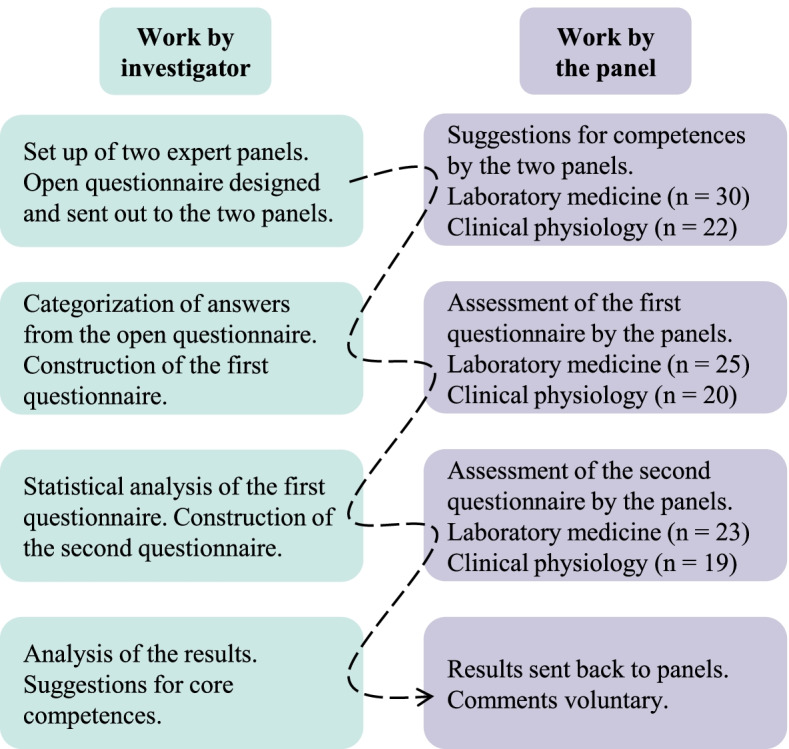
Overview of the Delphi process in the present study

A modified Delphi technique previously applied by some of the authors [8, 9, 45, 46] and also based on other publications was used. We decided to use only two rounds of questionnaires after the qualitative round. In the past, it has been shown that modified forms of the technique are most often used because the response rate usually decreases for each survey [47, 48]. The study was performed from April to June 2021. Panel members were asked to express their views in three steps. An overview of the study is shown in Fig. 1.

### Setting

In Sweden, the requirements for a biomedical laboratory scientist degree are defined by the Higher Education Ordinance, while a license to practice is issued by the National Board of Health and Welfare. Today, the biomedical scientist program is a three-year university program that includes learning outcomes for the degree and a degree project of at least 15 credits [49].

### Study participants

Since the theory and the practical work of a biomedical laboratory scientist in laboratory medicine and clinical physiology differ, we recruited the panel from both disciplines separately. Consequently, an invitation letter was sent to selected representatives from the two disciplines, many of whom were working in southern Sweden. The snowball sampling approach was then used to find more panel members which were invited by phone or through in-person meetings. Based on previous experience and studies, we estimated that we needed at least 20 participants from each discipline. We identified 58 potential participants from laboratories in various areas (clinical physiology, nuclear medicine, neurophysiology, clinical chemistry, clinical microbiology, clinical pathology, clinical immunology and transfusion medicine, and biomedical research laboratories) where biomedical laboratory scientists work or might seek employment. A total of 52 accepted and thereafter received an information letter and provided formal consent. An overview of the panel participants is presented in Table 1.

**Table 1:** Overview of panel participants. Number (percent)

Characteristics	Laboratory medicine 30 (%)	Clinical physiology 22 (%)
Women	24 (80)	16 (73)
Senior position	10 (33)	5 (23)
Experience >5 year	22 (73)	18 (82)
Licensed biomedical laboratory scientist	25 (83)	20 (91)

### Procedure

In the first round, all panel members were asked to list the competencies they considered necessary for a recently graduated biomedical laboratory scientist in a five-year perspective. Two of the authors (GE and TE) independently performed an inductive content analysis of the suggested competencies [50]. They identified and agreed on codes and categories. The codes were used to gather similar statements from the panel, and the codes were grouped into relevant categories (see Table 3). An example: the code communicate with colleagues gathered six statements and was finally phrased as (The recent graduates should be able to) meet and communicate with their colleagues. All codes were phrased as competencies resulting in a list of categorized competencies to be used as items in the questionnaire. This means that all items in the first questionnaire correspond to codes. In the second round panel members added a few competencies but these were not subjected to content analysis. Although we asked the panel members to express desired competencies, some also listed knowledge (e.g., knowledge in pathology, haematology, chromatography) or teaching formats (e.g., more laboratory exercises). These were coded and categorized but could not be phrased as competencies, but knowledge is always included in competencies.

The second round was a quantitative questionnaire comprising the competencies identified in round one. The panel members were asked to classify the competencies on a four-point scale (not necessary, useful, desirable, necessary) with an additional “cannot take a stand” option. They also had the opportunity to add new competencies for the third round. The third round was identical to the second but included the previous round’s results as the percentages of the participants choosing each score for each question. An example is given in Table 2. The identities of the panel members were known to the researchers only. The survey was conducted with the support system Survey and Report (Artisan, Sweden). The software handles anonymity and prevents a respondent from answering more than once. The participants were sent an email with a link to the survey, with two reminders sent automatically for each.

**Table 2:** Examples from the final survey

State the importance of the following competencies for a newly graduated biomedical laboratory scientist. The percentages indicate how the experts' answers were distributed in Survey 2					
Work in a quality-assured way	Not necessary, (0 %) □	Useful, (4 %) □	Desirable, (11 %) □	Necessary, (84 %) □	Stand (0 %) □
Help solve problems	Not necessary, (2 %) □	Useful, (18 %) □	Desirable, (71 %) □	Necessary, (9 %) □	Cannot take a stand (0 %) □
Use statistical methods and tools	Not necessary, (22 %) □	Useful, (44 %) □	Desirable, (18 %) □	Necessary, (13 %) □	Cannot take a stand (2 %) □
Perform ELISA and other immuno-chemistry	Not necessary, (2 %) □	Useful, (7 %) □	Desirable, (18 %) □	Necessary, (22 %) □	Cannot take a stand (51 %) □
Perform a spirometry	Not necessary, (4 %) □	Useful, (27 %) □	Desirable, (22 %) □	Necessary, (13 %) □	Cannot take a stand (33 %) □

### Consensus definition

Before starting this study, we defined consensus as 75 % of the panel participants agreeing that the competency was necessary in the final questionnaire [45, 51, 52]. We believed that necessary, specific competencies could be lost if they were essential to only one specific field. To avoid this, we labelled specific competencies for laboratory medicine and clinical physiology separately. This is also the reason as to why we added the response alternative “cannot take a stand”. This response alternative was thus not included as a denominator in the consensus calculations.

## Results

The expert panel consisted of 30 members from clinical laboratories focusing on laboratory medicine and 22 from clinical laboratories focusing on clinical physiology. The response rate after the third survey was 77 % for laboratory medicine and 86 % for clinical physiology. An overview of the number of responses for each round is given in Fig. 1. The content analysis resulted in eight categories; the number of competencies in each category after the first and third (consensus) round is presented in Table 3. A complete list of the 123 different competencies is shown in Additional file 1.

**Table 3:** Categories defined after content analysis

Categories	Number of competencies after	
	First round	Third round
Generic competencies	19	10
Hygiene and safety	7	5
Communication, collaboration, and colleagues	6	2
Scientific competence	9	0
Language	6	2
Analysis and assessment	16	5
Patient and ethics	5	4
Carrying out laboratory work and specific examinations	65	7
Total	123	35

For the third round, eight new competencies were suggested by panel members, but none reached consensus. In the third round, 25 of the 123 competencies were considered necessary by at least 75 % of the panel members (Table 4). This was an increase of 13 competencies compared to round two. When analysing the answers from the areas of laboratory medicine and clinical physiology separately, a further eight and two competencies reached the consensus level, respectively. The result for clinical physiology should be compared to round two, where only one competence reached the consensus level. The competencies identified in the separate analysis are more method-specific, which is to be expected given the differences between the specializations. In total, the consensus increased for 113 of the 115 competencies from round two to round three, even if not all of them reached the consensus level defined in this study.

**Table 4:** Competencies considered necessary according to our definition by the panel members. Data are presented as percentage of participants scoring necessary, divided by participant taking a stand, total, and separate disciplines. The number of participants taking a stand is shown in brackets

	All (*n*=42)	Clinical physiology (*n*=19)	Laboratory medicine (*n*=23)
Apply principles of quality assurance	100% (42)	100% (19)	100% (23)
Comply with confidentiality regulations	100% (42)	100% (19)	100% (23)
Follow the rules and guidelines in the workplace	98% (42)	95% (19)	100% (23)
Comply with basic hygiene rules	98% (42)	95% (19)	100% (23)
Apply General Data Protection Regulation	98% (42)	100% (19)	96% (23)
Consider patient safety, also in stressful situations	98% (42)	100% (19)	96% (23)
Communicate orally in Swedish	98% (42)	100% (19)	96% (23)
Respect working hours and booked appointments	95% (42)	95% (19)	96% (23)
Take responsibility for their work, discover and admit their own mistakes and report deviations	95% (42)	100% (19)	91% (23)
Accept guidance and support as needed	95% (42)	95% (19)	96% (23)
Use protective equipment if necessary	95% (42)	89% (19)	100% (23)
Read and write texts in Swedish	95% (42)	100% (19)	91% (23)
Keep the patient in focus and treat everyone in an ethical and respectful way	95% (41)	95% (19)	95% (22)
Communicate with the patient in a straightforward way	89% (37)	95% (19)	83% (18)
Show insight into the risks and consequences of their actions	88% (42)	100% (19)	78% (23)
Meet and communicate with their colleagues	86% (42)	79% (19)	91% (23)
Show respect and sensitivity in communication	86% (42)	79% (19)	91% (23)
Use common computer programs (word processing, calculation, search functions)	83% (36)	79% (18)	87% (18)
Perform examinations in a correct and specific manner depending on the patient and medical history	83% (42)	89% (19)	78% (23)
Prepare reagents, solutions, buffers, dilutions, and the like, and perform the necessary calculations.	81% (32)	44% (9)	96% (23)
Act professionally in common emergencies	81% (42)	79% (19)	83% (23)
Read professional literature in Swedish	81% (42)	84% (19)	78% (23)
Be aware of the consequences of analysis results for patients now and in the future	79% (39)	78% (18)	81% (21)
Show insight into how preanalytics affects results of analyses	79% (24)	50% (4)	85% (20)
Handle a sample and perform different types of analyzes	79% (33)	80% (10)	78% (23)
Show insight into the importance of traceability	71% (42)	84% (19)	61% (23)
Show insights about the sources of error in method and how these can affect the results	71% (42)	84% (19)	61% (23)
Distinguish normal findings from pathological and make reasonable assessments	63% (38)	83% (18)	45% (20)
Show flexibility and adaptability	64% (42)	79% (19)	52% (23)
Conduct an ECG examination	62% (26)	78% (18)	25% (8)
Practice care based on the patient's needs and codes of ethics	74% (34)	76% (17)	71% (17)
Apply sterile technique	74% (31)	75% (12)	74% (19)
Know which tubes belong to the referral and which sampling rules apply to the current analysis	66% (32)	75% (12)	60% (20)
Use centrifuges	73% (30)	38% (8)	86% (22)
Handle scales	60% (30)	13% (8)	77% (22)

## Discussion

This study resulted in 25 core competencies for the recently graduated biomedical laboratory scientist, with a panel member consensus score reaching the predefined level of consensus. A further ten were added as the consensus scores of panel members from clinical physiology (8) or laboratory medicine (2) did not coincide. It is interesting that of these 35 competencies 23 were generic. There is nothing in these that could distinguish them from generic competencies for any other health sciences profession. In the core curriculum for pharmacists and ambulance nurses, also developed by some of us [8, 46], there were many generic competencies, although not as dominating as in the present results. As seen in Table 4, the main core competencies in this study relate to quality, quality assurance, and accuracy, as well as different aspects of safety, respect, and trustworthiness (towards patients/clients and colleagues), including communication. The results also show the importance of self-awareness and professionality. The listed competencies are well in line with previously internationally documented competencies and the international ethical codes for the profession [12, 53]. This suggests that the results from the study are valid and that the international documents are reflected and have legitimacy in Sweden. However, the international guidelines [12] also list specific subjects and related methodological skills. In contrast, only a few competencies that reached consensus in the present study were directly connected to laboratory skills. When present, they only reached consensus within either laboratory medicine or clinical physiology. This is probably due to methodological differences as the IFBLS study, to our knowledge, did not use the Delphi technique.

None of the scientific competencies reached consensus in this study, which might be a bit surprising since there are publications discussing the future importance of critical and analytic thinking in the professions of laboratory medicine and management [54–56]. This might be explained by the fact that most of our panel members were biomedical laboratory scientists working in routine hospital-based laboratories. It might also be explained by the fact that the biomedical laboratory scientist education does not have a long university background, and there is still not total agreement on what defines this rather recent health profession. Until 1993, it was not a common degree or profession in Sweden, but rather scattered among at least five different professions. Indeed, it took until 2006 before it became a licensed profession. This is in contrast to pharmacy, which has been a recognised profession for hundreds of years. In our previous study on core competencies for pharmacists [8], some competencies in the scientific competency category qualified, e.g., evidence-based medicine, and have a critical approach. It is possible that the time-honoured pharmacy profession has established a common ground of both professional and scientific competencies. It is also likely that medical professionals would include at least evidence-based medicine if they were asked about core competencies. We are not aware of similar studies concerning other health science professions, such as nurses, physiotherapists, and occupational therapists, included in university level education only a few decades ago. It would be interesting to know if scientific competencies have been established as necessary in these professions.

When the present results are compared to the core competencies for biomedical laboratory scientists identified by Edgren in 2006 [9], the results are strikingly similar when differences in the application of the method are disregarded. In the previous study, the level of detail in the expression of the competencies was much higher. A few differences could be noted, however. In the present study quality assurance is directly mentioned, probably as a result of a growing use of this concept. Being able to use generic computer programs is also present, probably reflecting the increased use of digital devices by all staff. Two interesting competencies in the previous study are missing in the present: “have an understanding of the professional role of the biomedical scientist and how it relates to other health professions” and “have such knowledge in the biomedical field that in depth studies can be undertaken in a chosen subject”. Perhaps, they reflected that the common profession was rather new and that the formal education had become a three-year bachelor level programme. A possible explanation could be that over the fifteen years that have passed between the studies the missing competencies have become axiomatic elements of the profession.

The study was limited to regional circumstances as the experts were selected from the authors’ network of contacts. Care was taken during the study to select experts with diverse years of experience, gender, and from across all different disciplines. As two groups of experts were few in number, we cannot exclude the possibility of there being variations. Furthermore, although the response rate of the experts was high, it might not be representative of the whole cohort. A further weakness of a study of needed competencies in a five-year perspective is that it is based on current knowledge. When planning or revising an all-inclusive bachelor level laboratory medicine program, this Delphi study is only one of the sources one must consider. To reach a complete and relevant syllabus, the following must be consulted: national laws for education and medicine, ethical guidelines, and other publications that outline key qualities of future biomedical laboratory scientists based on the important role that this profession has in medicine today [57].

Previous studies have shown that in the future the functional specifications for biomedical laboratory scientists will evolve to include both deeper knowledge and new skills [54–56]. This concerns deeper knowledge in new advanced laboratory methods and new skills connected to the growth of evidence-based laboratory medicine where there is a demand to choose the most convenient methods with respect to diagnostic, medical, ethical, economic, and societal aspects. These changes can be accommodated within the broad scope of biomedical laboratory science, provided that educational efforts are intensified and that advanced level education is sizably expanded. A further focus on evidence-based practice and on specialized education following a BSc-degree in laboratory medicine will also be a demand. In addition, life science and the medicine of tomorrow would gain much to implement, encourage, and put resources into master level programmes and doctoral studies for biomedical laboratory scientists.

## Conclusions

In our study, which used a modified three-round Delphi technique process, 52 experts reached consensus in identifying core competencies that could be transferred to learning outcomes for a revised biomedical laboratory science BSc-degree. This is important in the development of a degree that is attractive both to students and to future employers. The main results from this Delphi study show the importance of generic competencies that relate to quality, safety, respect, trustworthiness, communication, self-awareness, and professionality for future biomedical laboratory scientists. Some differences in competences that reached consensus between clinical physiology and laboratory medicine were discovered, whereof most could be coupled to different methods for the respective discipline. The results can be used as learning outcomes in a competency-based education with complementary information from other sources, guidelines, laws, and scientific publication to provide students with all the competencies needed to work as a professional biomedical laboratory scientists.

## Supplementary Information

**Additional file 1.**

## Data Availability

The datasets generated and analysed during the current study are available upon reasonable request with the corresponding author.
