# Supplementary material for: Core competencies for a biomedical laboratory scientist – a Delphi study
Source: BMC Med Educ. 2022 Jun 20;22:476. doi: 10.1186/s12909-022-03509-1 (PMC9208704; doi:10.1186/s12909-022-03509-1)
Supplement: Supplementary file 1 — Additional file 1. [file 12909_2022_3509_MOESM1_ESM.docx]

**Additional file 1**

A complete list of the 123 different competencies suggested by the panel members after the first round. Data are presented as percentage of participants scoring necessary, divided by participant taking a stand, total, and separate disciplines in round 3.

|  | All  (n=42) | Clinical Physiology (n=19) | Laboratory Medicine (n=23) |
| --- | --- | --- | --- |
| Apply principles of quality assurance | 100% | 100% | 100% |
| Comply with confidentiality regulations | 100% | 100% | 100% |
| Follow rules and guidelines in the workplace | 98% | 95% | 100% |
| Comply with basic hygiene rules | 98% | 95% | 100% |
| Apply General Data Protection Regulation | 98% | 100% | 96% |
| Consider patient safety, also in stressful, situations | 98% | 100% | 96% |
| Communicate orally in Swedish | 98% | 100% | 96% |
| Respect working hours and booked appointments | 95% | 95% | 96% |
| Take responsibility for their work, discover and admit their own mistakes and report deviations | 95% | 100% | 91% |
| Accept guidance and support as needed | 95% | 95% | 96% |
| Use protective equipment if necessary | 95% | 89% | 100% |
| Read and write texts in Swedish | 95% | 100% | 91% |
| Keep the patient in focus and treat everyone in an ethical and respectful way | 95% | 95% | 95% |
| Communicate with the patient in an easy and clear way | 89% | 95% | 83% |
| Show insight into the risks and consequences of their own actions | 88% | 100% | 78% |
| Meet and communicate with their colleagues | 86% | 79% | 91% |
| Show respect and sensitivity, in communication | 86% | 79% | 91% |
| Use common computer programs (word processing, calculation, search functions) | 83% | 79% | 87% |
| Perform examinations in a correct and specific manner depending on the patient and medical history | 83% | 89% | 78% |
| Prepare reagents, solutions, buffers, dilutions and the like, and be able to perform the necessary calculations | 81% | 44% | 96% |
| Act professionally in common emergency situations | 81% | 79% | 83% |
| Read professional literature in Swedish | 81% | 84% | 78% |
| Be aware of the consequences of analysis results for patients now and in the future | 79% | 78% | 81% |
| Show insight into how pre-analytics affects results of analyses | 79% | 50% | 85% |
| Handle a sample and perform different types of analyses | 79% | 80% | 78% |
| Apply sterile technique | 74% | 75% | 74% |
| Practice care based on the patient's needs and codes of ethics | 74% | 76% | 71% |
| Use centrifuges | 73% | 38% | 86% |
| Show insight into the importance of traceability | 71% | 84% | 61% |
| Show insights about the sources of error in methods and how these can affect the results | 71% | 84% | 61% |
| Know which tubes belong to the referral and which sampling rules apply to the current analysis | 66% | 75% | 60% |
| Assess the quality of a (patient) sample and whether it is suitable for analysis e.g. hemolysis, coagulation or ageing | 65% | 50% | 71% |
| Distinguish acute samples from routine samples | 65% | 50% | 74% |
| Show flexibility and adaptability | 64% | 79% | 52% |
| Distinguish normal findings from pathological and make reasonable assessments | 63% | 83% | 45% |
| Show insight into common concepts such as lipemia, hemolysis, jaundice, interference, density, coating | 62% | 38% | 71% |
| Conduct an ECG examination | 62% | 78% | 25% |
| Perform pipetting and calibrate pipettes | 61% | 33% | 73% |
| Handle scales | 60% | 13% | 77% |
| Adapt to a new workplace and to changes | 60% | 63% | 57% |
| Use calibration and controls insightfully | 59% | 47% | 68% |
| Call referrers and request additional information | 58% | 42% | 71% |
| Be able to read, handle, register, and interpret referrals | 56% | 53% | 59% |
| Take personal responsibility for the method used for an examination | 55% | 53% | 58% |
| Communicate and collaborate in teams with different skills and professions | 52% | 58% | 48% |
| Accept feedback from colleagues | 50% | 47% | 52% |
| Perform various analyses e.g. Hb measurement, ABO-, and RhD determination | 48% | 22% | 67% |
| Assess compatibility of blood to be transfused and show understanding of potential problems. | 44% | 20% | 55% |
| Measure blood pressure | 42% | 61% | 20% |
| Perform an overall interpretation of an ECG, and distinguish a normal result from a deviant one | 42% | 56% | 13% |
| Explain and conduct examinations professionally and ethically for patients and relatives | 41% | 37% | 46% |
| Perform ELISA and other immunochemistry techniques | 40% | 20% | 47% |
| Take personal responsibility for the examination regarding the form of response | 40% | 22% | 59% |
| Use the equipment available in the laboratory in question | 39% | 37% | 41% |
| Work under stress with maintained concentration | 38% | 37% | 39% |
| Be able to perform biochemical analyses | 36% | 0% | 50% |
| Perform microscopy and assess, e.g. blood cells in blood smears and urine sediments | 36% | 14% | 44% |
| Deliver blood and show understanding for possible consequences | 35% | 33% | 36% |
| Seek information about different analyzes and identify critical elements in each analyses | 35% | 36% | 35% |
| Perform an exercise ECG and apply criteria for interruption | 33% | 39% | 17% |
| Carry out risk analysis and use applicable protective measures | 32% | 39% | 26% |
| Identify their strengths and limitations and their need for additional knowledge | 31% | 47% | 17% |
| Perform standard laboratory examinations | 28% | 32% | 23% |
| Grow patient samples on culture media | 28% | 25% | 30% |
| Perform blood sampling (venous and capillary) | 27% | 25% | 28% |
| Perform various chromatographic analyzes such as HPLC, GC | 26% | 20% | 29% |
| Perform electrophoresis | 25% | 17% | 29% |
| Perform fluorescence-based techniques | 24% | 17% | 27% |
| Read professional literature in English | 24% | 26% | 22% |
| Find relevant information and sources of knowledge and interpret those critically | 24% | 21% | 26% |
| Handle laboratory dishes | 23% | 25% | 23% |
| Be able to work with cell cultures | 22% | 0% | 31% |
| Perform flow cytometry | 22% | 0% | 29% |
| Show insights about different types of culture media and incubation environments and why they are used | 22% | 14% | 27% |
| Be involved in their own competence development | 21% | 21% | 22% |
| Show insight into their own and others' role in the team | 21% | 21% | 22% |
| Insert PVK (peripheral venous catheter) | 21% | 31% | 8% |
| Conduct an EEG examination | 21% | 23% | 17% |
| Be able to compile results and present them in a scientific and clear way | 21% | 17% | 24% |
| Be able to work with pharmacokinetic analyses | 20% | 0% | 29% |
| Use all kinds of chemicals | 20% | 29% | 17% |
| Provide feedback to colleagues | 19% | 16% | 22% |
| Perform testing of antibiotic resistance | 19% | 0% | 30% |
| Take samples such as fungal scrapes, perform bacterial- and virus culture | 19% | 17% | 20% |
| Read and write texts in English | 17% | 21% | 13% |
| Conduct examinations in traditional clinical physiology | 17% | 17% | 17% |
| Connect EMG and measure nerve conduction velocity | 17% | 15% | 20% |
| Perform ultrasound examinations for the heart and vessels. | 16% | 17% | 14% |
| Handle daily maintenance and perform simple troubleshooting of instruments | 15% | 16% | 14% |
| Insightfully implement evidence-based knowledge | 13% | 17% | 10% |
| Evaluate images where radiopharmaceuticals have been used | 13% | 13% | 14% |
| Independently write and sign statements on examinations | 13% | 5% | 25% |
| Perform ultrasound examinations | 13% | 12% | 14% |
| Use isotopes and manage risks in connection with their use | 13% | 13% | 11% |
| Interpret statistical results | 12% | 11% | 13% |
| Perform examinations usin nuclear medicine techniques | 12% | 11% | 14% |
| Perform spirometry | 12% | 11% | 14% |
| Work for improvement and increased efficiency | 12% | 0% | 22% |
| Manage commonly use IT-based analysis software and be able to solve problems with the instruments analyses | 10% | 6% | 14% |
| Be able to work in projects of various forms | 10% | 0% | 19% |
| Show insights into limitations in technical equipment | 10% | 5% | 14% |
| Communicate orally in English | 10% | 5% | 13% |
| Contribute to problem solving | 10% | 0% | 17% |
| Supervise students and new colleagues | 10% | 11% | 9% |
| Use up-to-date equipment, including hybrid modalities (e.g . X-ray and MRI) | 8% | 6% | 13% |
| Manage water facilities | 8% | 0% | 11% |
| Perform turbidometry | 8% | 0% | 11% |
| Critically review scientific literature | 7% | 5% | 9% |
| Use statistical methods and tools | 7% | 11% | 4% |
| Plan and participate in project work | 7% | 5% | 9% |
| Show a critical attitude to experimental results | 5% | 6% | 5% |
| Perform searches in databases | 5% | 6% | 4% |
| Perform magnetic resonance imaging | 5% | 0% | 20% |
| implement hybrid modalities | 5% | 0% | 20% |
| Register chemicals in Klara. | 4% | 0% | 5% |
| Master interview techniques | 3% | 6% | 0% |
| Show insights into how research methodology and equipment can affect research projects | 3% | 6% | 0% |
| Be able to lead project work | 3% | 0% | 5% |
| Work in an accredited laboratory | 2% | 6% | 0% |
| Contribute to the development of health care | 2% | 0% | 4% |
| Plan and carry out major studies that are ongoing over a longer period of time and has different endpoints | 0% | 0% | 0% |
| Speak and understand languages ​​other than Swedish and English | 0% | 0% | 0% |
| Perform x-ray examinations | 0% | 0% | 0% |
